# A native annual forb locally excludes a closely related introduced species that co-occurs in oak-savanna habitat remnants

**DOI:** 10.1093/aobpla/plaa045

**Published:** 2020-08-25

**Authors:** Jens C Johnson, Jennifer L Williams

**Affiliations:** Department of Geography and Biodiversity Research Centre, University of British Columbia, Vancouver, BC, Canada

**Keywords:** Coexistence, determinants of plant community diversity and structure, environmental heterogeneity, invasion ecology, plant competition, plant population and community dynamics

## Abstract

Despite the ubiquity of introduced species, their long-term impacts on native plant abundance and diversity remain poorly understood. Coexistence theory offers a tool for advancing this understanding by providing a framework to link short-term individual measurements with long-term population dynamics by directly quantifying the niche and average fitness differences between species. We observed that a pair of closely related and functionally similar annual plants with different origins—native *Plectritis congesta* and introduced *Valerianella locusta*—co-occur at the community scale but rarely at the local scale of direct interaction. To test whether niche and/or fitness differences preclude local-scale long-term coexistence, we parameterized models of competitor dynamics with results from a controlled outdoor pot experiment, where we manipulated densities of each species. To evaluate the hypothesis that niche and fitness differences exhibit environmental dependency, leading to community-scale coexistence despite local competitive exclusion, we replicated this experiment with a water availability treatment to determine if this key limiting resource alters the long-term prediction. Water availability impacted population vital rates and intensities of intraspecific versus interspecific competition between *P. congesta* and *V. locusta*. Despite environmental influence on competition our model predicts that native *P. congesta* competitively excludes introduced *V. locusta* in direct competition across water availability conditions because of an absence of stabilizing niche differences combined with a difference in average fitness, although this advantage weakens in drier conditions. Further, field data demonstrated that *P. congesta* densities have a negative effect on *V. locusta* seed prediction. We conclude that native *P. congesta* limits abundances of introduced *V. locusta* at the direct-interaction scale, and we posit that *V. locusta* may rely on spatially dependent coexistence mechanisms to maintain coexistence at the site scale. In quantifying this competitive outcome our study demonstrates mechanistically how a native species may limit the abundance of an introduced invader.

## Teaser

We observed that closely related annual plants with different origins—native *Plectritis congesta* and introduced *Valerianella locusta*—co-occur at community scales but rarely at the scale of direct interactions. We parameterized models of competitor dynamics to quantify niche and average fitness differences between these species with results from an experiment where we manipulated competitor densities and water availability. Our model, corroborated with field data, predicts that *P. congesta* competitively excludes *V. locusta* in direct competition across water availability conditions. In quantifying this outcome our study demonstrates mechanistically how a native species may limit abundance of an introduced invader.

## Introduction

Introduced plant species are ubiquitous features of contemporary ecosystems that negatively impact biodiversity and ecosystem function (Vilà *et al.* 2011; Vellend *et al.* 2013). Despite the widespread occurrence of introduced species in natural systems, a firm understanding of the mechanisms that underlie invasion success and impact is still lacking. From a coexistence theory perspective (Chesson 2000), introduced species are expected to more successfully establish and spread when they either possess functional differences that reduce competition with native species for limiting resources—i.e. stabilizing niche differences—or when they possess functional differences that provide a relative competitive advantage for acquiring limiting resources over native species—i.e. average fitness differences (Shea and Chesson 2002; Melbourne *et al.* 2007; Godoy and Levine 2014). Niche and fitness differences simultaneously influence the impact that introduced species have on native species as they invade a system. Invading species will have low impact on the native community when they possess a high niche difference (open niche space that enables establishment) coupled with little or no invader fitness advantages (small competitive impact on native neighbours). The most detrimental invasions should occur when invaders possess high average fitness and low niche differences versus the native residents (MacDougall *et al.* 2009; Lai *et al.* 2015). Since species with close phylogenetic relationships and high trait overlap are predicted to have relatively high niche overlap (HilleRisLambers *et al.* 2012), introduced species that are closely related to native residents have the potential to have high impacts if they also have high average fitness differences.

Modern coexistence theory provides a framework to directly quantify niche and average fitness differences (Chesson 2000) by extrapolating demographic rates of competing species in small-scale empirical studies to population-level predictions (e.g. Adler *et al.* 2010; Godoy and Levine 2014; Kraft *et al.* 2015). Recently, this perspective has been used to explain the impacts of introduced plant species on members of native plant communities. Coexistence between species in mixed native and non-native plant communities appears to be best predicted by functional traits and patterns, e.g. phenology window in a study of a California annual plant community (Godoy *et al.* 2014) rather than native versus non-native origin (Godoy *et al.* 2014) or phylogenetic distance (Germain *et al.* 2016). In fact, fitness differences can rapidly accumulate between pairs of introduced and native species, unrelated to the accumulation of niche differences over time (Germain *et al.* 2016).

In this study, we used a coexistence framework to evaluate the impact of an introduced species, *Valerianella locusta* (Caprifoliaceae), on a closely related native species, *Plectritis congesta* (Caprifoliaceae). *Valerianella locusta* and *P. congesta* are annual forbs that share similar growth forms and life histories, and currently overlap in range in western North America where *P. congesta* is native and where *V. locusta* was introduced from Eurasia (Consortium of Pacific Northwest Herbaria 2017). Across coastal grassland communities, *V. locusta* currently occurs at relatively similar frequency to its native analogue, and these species co-occur at the scale of remnant habitat patches (MacDougall and Turkington 2005). However, we observed that these common species rarely co-occur at the scale of direct interaction, a pattern within communities that has not yet been quantified.

Empirical results from numerous studies highlight that interaction strengths (Reader and Best 1989; Adler *et al.* 2012), intrinsic growth rates (Reader and Best 1989; Suttle *et al.* 2007; Tenhumberg *et al.* 2018) and predictions for coexistence (e.g. Bimler *et al.* 2018) can be specific to the set of environmental conditions in which they are measured. A suite of recent studies that manipulate the availability of limiting resources and link results from these short-term experimental treatments to long-term population-level predictions demonstrate that limiting resource availability can shift coexistence predictions, even amongst species pairs with functional overlap that may be expected to have similar strategies for resource uptake and use (Germain *et al.* 2016; Bimler *et al.* 2018; Germain *et al.* 2018; Matias *et al.* 2018; Wainwright *et al.* 2019). Changes in limiting resource availability may alter the competitive outcome if physiological or stoichiometric trade-off advantages for accessing a shared limited resource pool are eliminated by resource additions (Tilman 1982; Harpole and Tilman 2007; HilleRisLambers *et al.* 2012), or by altering niche overlap via plastic phenotypic responses (Turcotte and Levine 2016). Mathematically, when species are similar and niche differences are low (such as we expect with *P. congesta* and *V. locusta* in our system), only very small changes in fitness difference could enable a coexistence shift (Chesson 2000). In the Mediterranean-climate grassland habitats where *P. congesta* and *V. locusta* co-occur, water availability is a key limiting resource and varies at the scale of metres (MacDougall and Turkington 2005; Lea 2006). Because limiting resources may change coexistence predictions, variation in water availability may shift the long-term competitive outcome between *P. congesta* and *V. locusta* in some spatial patches within a habitat remnant.

To determine how competitive interactions between native *P. congesta* and introduced *V. locusta* mediate *V. locusta* invasion impact and success, we addressed the following questions: (i) Are abundances of *V. locusta* and *P. congesta* negatively related at the direct-interaction scale? (ii) Are *V. locusta* and *P. congesta* predicted to coexist at the scale of direct interactions? Moreover, given the potential for water availability to drive environmentally dependent coexistence as well as the importance of water as a spatially variable limiting resource in our system, is this competitive outcome dependent on water availability? (iii) Do demographic measures from the field support the experimental results and demographic predictions of long-term competitive outcome?

Because we expected niche overlap between these species to be relatively high and observed that they rarely co-occur at the scale of direct interaction despite regular community co-occurrence, we hypothesized that a fitness inequality—expected to accumulate over evolutionary time—would outweigh niche differences and allow one species to competitively exclude the other at the scale of direct interaction. We hypothesized that within-community spatial variation in abiotic conditions could alter the interaction strengths and intrinsic growth rates of *P. congesta* and *V. locusta*. If this hypothesis were supported, a spatial storage effect could ultimately enable these species to coexist at the community scale by allowing the generally inferior competitor to coexist or have a competitive advantage in certain environmental patches (Chesson 1994).

## Materials and Methods

### Study species and study region

The Garry oak savanna ecosystem in south-western British Columbia is part of a climate and vegetation complex that extends south along Pacific lowlands towards California, with a sub-Mediterranean climate. Local-scale environmental heterogeneity including soil depth, soil moisture and shade cover, drive strong local variability in water availability at the scale of metres or finer (MacDougall and Turkington 2005; Vellend *et al*. 2008). Vegetation is dominated by grasses and a diverse community of early-spring flowering herbaceous plants (Vellend *et al*. 2008). Native flora now typically constitute <10 % of all species cover (Trowbridge *et al.* 2017).

The forb *P. congesta* (sea blush) is native and locally common in Garry oak communities. *Plectritis congesta* is a winter annual that lacks dormancy and typically germinates in the fall or early spring, flowers between March and June and then sets seed and dies by mid-summer (Ganders *et al.* 1977; Young-Mathews 2012). Along with *P. congesta*, *V. locusta* (corn-salad) belongs to the Valerianoideae subfamily of Caprifoliaceae (Bell and Donoghue 2005) and exhibits a similar growth form and phenology (Ernet 1977; Jacobs *et al.* 2010). *Valerianella locusta* was introduced to the Pacific Northwest region of North America as early as the 1880s (Consortium of Pacific Northwest Herbaria 2017) and has spread throughout the Garry oak ecosystem where it is now locally common (MacDougall and Turkington 2005).

*Valerianella locusta* could be considered a low-impact invader given that it occurs relatively infrequently compared to more aggressive and ubiquitous introduced species in these habitats, such as the large non-native perennial grasses that dominate the Garry oak ecosystem (MacDougall and Turkington 2005). We do not expect ruderal *V. locusta* to drive large negative impacts on the plant community as a whole. But we expected that the predicted high level of direct interaction between this focal species pair combined with their general pattern of co-occurrence provides an ideal opportunity to explore interactions between environmental variation and competition and the effects of this interaction on long-term invasion outcome.

### Field surveys

To compare results from an experiment garden (details in next section) with field conditions and plant performance, we surveyed *P. congesta* and *V. locusta* at two sites on south-eastern Vancouver Island, BC: Nature Conservancy of Canada Cowichan Garry Oak Preserve and Stoney Hill Regional Park. To investigate correlations in abundance at two spatial scales, we quantified plant abundance, collected demographic data and evaluated environmental conditions in 1-m^2^ plots (*n* = 70), and additionally, in two 0.1-m^2^ plots nested within each 1-m^2^ plots (*n* = 140). We counted the number of *P. congesta* and *V. locusta* per plot, and for 10 individuals of each species in each 0.1-m^2^ plot (or as many individuals as possible if there were fewer than 10), we quantified number of inflorescences, plant height and main inflorescence size to use as proxies for fecundity. Using a marked measuring rod, we categorically classified soil depth in the bottom-right and top-left corners of each plot as either <12 cm or >12 cm. We used a soil moisture probe (FieldScout TDR-300, Spectrum Technologies Inc.) to measure percent volumetric water content (%VWC) at six points in each 1-m^2^ plot (two points in each nested 0.1 m^2^ and two additional points at opposite corners of the 1-m^2^ plot) at depths of 7.6 and 12 cm. We did not test soil moisture if the soil depth was <7.6 cm.

### Resource competition experiment

To determine whether these two closely related species can locally coexist, we set up an outdoor experiment in pots (7.6-litre, 23-cm-diameter) filled with potting soil (Sunshine Mix #1, Terralink), growing *P. congesta* and *V. locusta* in density gradients of conspecific or heterospecific neighbours. From this experiment, we quantified two demographic vital rates to parameterize population models: density-independent germination rates (*g*) and density-dependent seed production (*F*). Given high germination rates and seed bank mortality (Saanich Native Plants, pers. comm.), our model assumes that there is no opportunity for a temporal storage effect.

To evaluate species’ germination rates (*g*), we sowed and monitored 432 seeds of each species (72 pots per species with 3 seeds per pot). Germination rates were determined in April 2017, 32–36 days after sowing, by counting the number of seeds that had successfully germinated and survived. Pots were kept outside on the University of British Columbia campus, Vancouver, BC, and allowed to experience natural environmental and weather conditions during the germination portion of the experiment.

To determine density-dependent seed production (*F*), we removed all but one successful germinant from the centre of the 23-cm-diameter pots and treated this as a focal individual. We had also sown and then thinned neighbours to match one of four neighbour density levels: (i) 16 pots with no neighbours; (ii) 12 pots with four heterospecific neighbours and 12 pots with four conspecific neighbours; (iii) eight pots with 10 heterospecific neighbours and eight pots with 10 conspecific neighbours; and (iv) eight pots with 20 heterospecific neighbours and eight pots with 20 conspecific neighbours **[see****Supporting Information—Fig. S1****]**. Larger sample sizes for low neighbour densities were selected due to higher expected variation in low-density population growth rate. Densities were chosen to bracket natural densities observed in the field (Cowichan Garry Oak Preserve, Duncan, BC, C. Trowbridge, unpubl. data). To obtain values of *F* we collected seeds from focal individuals at the end of the growing season by removing seeds as they matured and counted seeds by hand in the lab.

To explore whether the average fitness and niche differences depended on water availability, we replicated the density gradient under water treatment regimes. As natural rainfall decreased over the course of the experiment, we provided ample water to one treatment group (hereafter, the wet treatment) and decreased watering regularity and duration in the other (hereafter, the dry treatment). Additionally, we erected a 30 % shade cloth for the last 2 weeks of the experiment over plants in the wet treatment to reduce moisture loss. Shade may alter other conditions such as temperature and CO_2_ concentration which influence plant growth (Song *et al.* 2012), and may confound the effects of water availability. However, the additional shade mimics field conditions, where patchy savanna tree cover ameliorates evaporation, and thus is relevant to creating wet versus dry environmental treatments. **See****Supporting Information—Methods** for detailed growing procedures.

### Analysis of field data

We used data from the field surveys of the abundances *P. congesta* and *V. locusta* to evaluate whether there was a negative relationship between species abundances at both the 0.1-m^2^ scale and 1 m^2^ using generalized linear models (GLMs) with negative binomial error distribution, including random effects for transect and site to account for spatial correlation using *lme4* in R (Bates *et al.* 2015).

To test our expectation that these plants exhibit an interaction between competition and environmental variation in the field, we first fit GLMs with negative binomial distributions using glm.nb with *MASS* in R (Venables and Ripley 2002) to predict seed production of plants growing at field sites using seed counts from a sample of individuals alongside measurements of number of inflorescences, plant height and main inflorescence size. For *P. congesta*, we used seed counts and measurements from the untreated plants in a separate field pollination experiment; for *V. locusta*, we used seed counts and measurements from focal individuals in the pot experiment, pooled across all densities, because we lacked seed counts from the field site. To extrapolate these predictions to plants growing at both field sites, we selected the best models for each species by comparing Akaike information criterion (AIC) scores of all possible nested models and selecting more complicated models only when we observed a significant (>2) reduction in AIC score. The best model for *P. congesta* included only plant height as the predictor of seed count, while the best model for *V. locusta* included number of inflorescences, plant height and main inflorescence size **[see****Supporting Information—Table S2****]**. We then predicted seed production of plants in the field plots given the fitness proxies we measured.

With these predicted seed numbers as a response, we determined how intraspecific and interspecific densities impacted the fecundity of each species using GLMs with negative binomial error distribution and including random effects to account for spatial correlation with *lme4* in R (Bates *et al.* 2015). We evaluated whether *P. congesta* and *V. locusta* responded uniquely to environmental variation by determining whether soil moisture and soil depth differentially influenced the predicted seed production in *P. congesta* and *V. locusta*, again using GLMs with negative binomial error distribution and including random effects to account for spatial correlation with *lme4* in R (Bates *et al.* 2015). We used likelihood-ratio tests (LRTs) to compare the full mixed-effects GLMs to GLMs without these fixed effects. We also tested whether interactions between neighbour density and these environmental conditions were significant by comparing models with LRTs. If water availability plays a strong role in mediating the competition between these species, we expected to see interactions between neighbour density and water availability on plant reproduction in natural communities.

### Analysis of resource competition experiment

To determine whether *P. congesta* and *V. locusta* can coexist in direct competition, we used data on density-dependent seed production (*F*) and species’ germination rates (*g*) of each species from the assembled wet and dry treatment communities to parameterize Beverton–Holt annual plant population growth models (Chesson 2000; Godoy and Levine 2014). Our models estimate population growth rates in the absence of competition (λ), and the influence of neighbour density and identity on population growth rates (α _species *i*, species *i*_ for intraspecific competitive effects and α _species *i*, species *j*_ for interspecific competitive effects) for each species by water treatment combination **[see****Supporting Information—Equations 1****and****2****]**, using generalized non-linear regression with a negative binomial distribution with *gnlm* in R (Swihart and Lindsey 2019).

Demographic measurements of λ, α and *g* were then synthesized into estimates of the stabilizing niche (1 − ρ; **see****Supporting Information—Equation 3**) and fitness (κ _*j*_/κ _*i*_) differences that determine the outcome of competition (Chesson 2000; Godoy and Levine 2014). Niche overlap approaches a value of 1 (and niche difference, 1 − ρ, approaches a value of 0) when α _*ii*_ = α _*ij*_ and α _*jj*_ = α _*ji*_. The average fitness ratio between species is defined as the product of the demographic potential ratio **[see****Supporting Information—Equation 4****]** and the competitive response ratio [see **Supporting Information—Equation 5**] of *V. locusta* relative to *P. congesta***[see****Supporting Information—Equation 6****]**.

Niche and fitness differences are functions based on combinations of parameter estimates (λ, α, *g*) with associated uncertainties. We used the propagate function with *propagate* in R (Spiess 2018) to carry errors in the parameter estimates of λ and α to obtain confidence intervals around the estimates of niche and fitness difference. *Propagate* uses matrix algebra to calculate the propagated error of a function (given estimates and errors for the function inputs) by first-/second-order Taylor expansion accounting for covariance structure. Finally, we considered coexistence conditions satisfied when $\;\;\rho <\;\kappa_{j}/\kappa_{i}<\;\frac{1}{\rho }$, meaning that coexistence is most likely when niche overlap is low, that is, when ρ approaches 0, and the fitness ratio (κ _*j*_/κ _*i*_) is relatively even between species, that is, when κ approaches 1.

Coexistence can also be described using the low-density population growth rates of competing species; species are expected to coexist when both species can invade and increase in abundance in a patch dominated by the opposing species (MacArthur and Levins 1967; Chesson 2000). We quantified the invasion growth rate of species *j* (occurring at a diminishingly low density) invading species *i* (occurring at its equilibrium population density) (Hart *et al.* 2019) as:


$$\begin{array}{l} \lambda_{j}/\left(1+\;\alpha_{ji}\;*\;\frac{\left(\lambda_{i}-1\right)}{\;\;\;\alpha_{ii}}\right)\; \end{array}$$

All statistics and modelling were done in R, version 3.6.2 (R Development Core Team 2019).

## Results

### Interaction scale abundance patterns

**Figure 1.**
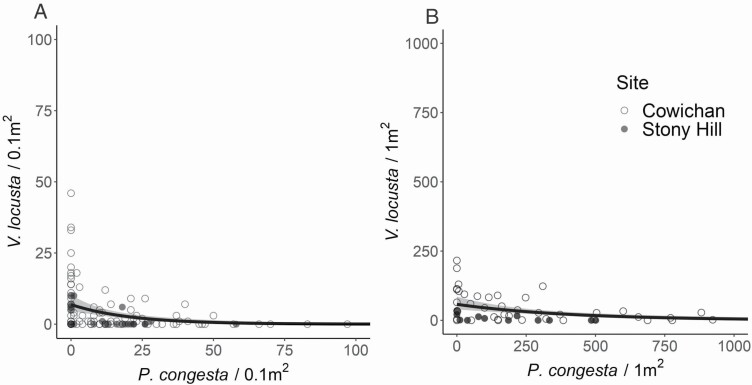
Relationships between *P. congesta* and *V. locusta* abundances in habitat remnants where they co-occur in (A) 0.1-m^2^ and (B) 1-m^2^ plots across two field sites in the Garry oak savanna (southern Vancouver Island, BC), with 95 % CIs.

In natural plant communities, we found that the abundances of *V. locusta* and *P. congesta* showed a negative relationship at both the 0.1-m^2^ scale ($\chi_{1}^{2}$ = 10.76, *P* < 0.01) (Fig. 1A) and at the 1-m^2^ scale ($\chi_{1}^{2}$ = 14.18, *P* < 0.01) (Fig. 1B).

### Resource competition experiment

**Figure 2.**
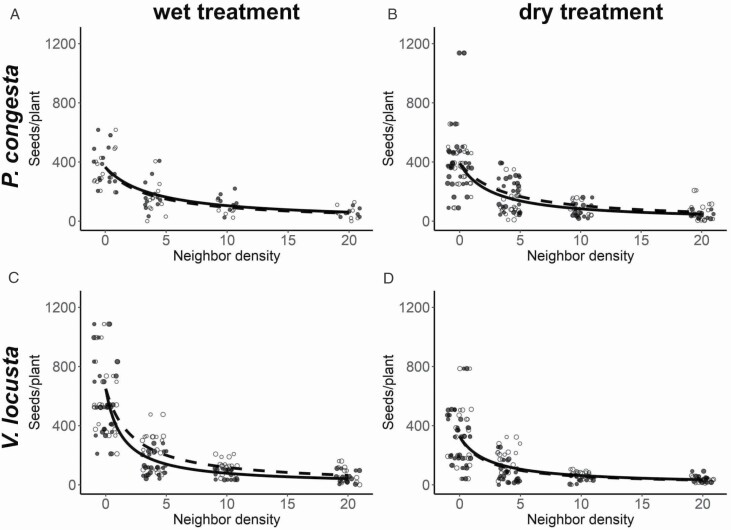
Density-dependent seed production of *P. congesta* in the dry treatment (A) and in the wet treatment (B) and seed production of *V. locusta* in the dry treatment (C) and in the wet treatment (D) are plotted against number of neighbour plants per growing pot. Seed production values against conspecific neighbours are denoted with filled circles and solid trend lines, while seed production values against heterospecific neighbours are denoted with open circles and dashed trend lines. The same set of individuals grown with no neighbours (neighbour density = 0) were used to fit relationships for both intraspecific and interspecific competition for each focal species by water treatment. Points were jittered for each neighbour density to improve legibility.

Intrinsic population growth rate (λ) of *P. congesta* did not significantly change between treatments, while λ of *V. locusta* was greater in the dry treatment versus the wet treatment (Table 1). Although the intraspecific interaction coefficients (α _*ii*_ and α _*jj*_) changed significantly between treatments for both species (water addition increased *P. congesta* intraspecific competition and reduced *V. locusta* intraspecific competition), intraspecific interaction coefficients were only significantly different from interspecific interaction coefficients for *V. locusta* in the dry treatment ((α _*V. locusta*, *V. locusta*_ = 0.73 (SE = 0.11); (α _*V. locusta*, *P. congesta*_ = 0.45 (SE = 0.05)) (Table 1; Fig. 2). Density-independent germination rates were high and similar between species, 67.0 % for *P. congesta* and 89.1 % for *V. locusta*.

**Table 1.** Estimates of density-dependent population growth rate parameters for the native *P. congesta* and introduced *V. locusta* from plants grown with wet and dry soil moisture treatments. Here, λ represents per-germinant fecundity in the absence of competition and α _*ij*_ represents an interaction coefficient, that is, the effect on species *i* by species *j.* For λ and α values, means and standard errors are given for estimates from 50 bootstrap samples of the density-dependent seed production data (Fig. 1). The demographic ratio is reported as *V. locusta*/*P. congesta* (a ratio > 1 indicates that *V. locusta* has a higher demographic potential). The competitive response ratio is reported as *P. congesta*/*V. locusta* (a ratio < 1 indicates that *P. congesta* is less sensitive to competition). The fitness ratio is reported as *V. locusta*/*P. congesta* (a ratio > 1 indicates that *V. locusta* has a higher average fitness). Coexistence is possible when niche overlap (ρ) is low, i.e. when ρ approaches 0, and the fitness ratio (κ) is relatively even between species, i.e. when κ approaches 1. Asterisks (*) indicate parameters that significantly changed between soil moisture treatments. Standard error estimates reported for ρ and κ were propagated from the sampled data used to estimate λ and α. **See****Supporting Information—Equations 2****–****7** for details on parameter estimation

	Dry treatment	Wet treatment
λ *P. congesta*	365.9 (17.7)	391.23 (27.8)
λ *V. locusta*	650.0 (57.4)*	327.4 (24.8)*
α (*P. congesta*, *P. congesta*)	0.25 (0.02)*	0.37 (0.04)*
α (*P. congesta*, *V. locusta)*	0.29 (0.03)	0.27 (0.07)
α (*V. locusta*, *V. locusta*)	0.73 (0.11)*	0.47 (0.06)*
α (*V. locusta*, *P. congesta*)	0.45 (0.05)	0.42 (0.04)
Niche overlap (ρ)	0.85 (0.10)	0.81 (0.16)
Demographic ratio	1.78 (0.18)*	0.84 (0.09)*
Competitive response ratio	0.47 (0.05)*	0.71 (0.14)*
Average fitness ratio (κ _*j*_/κ _*i*_)	0.83 (0.13)	0.60 (0.14)
Interaction outcome (coexistence predicted if: ρ < κ _*j*_/κ _*i*_ < 1/ρ)	*P. congesta* predicted to exclude *V. locusta*	*P. congesta* predicted to exclude *V. locusta*

**Figure 3.**
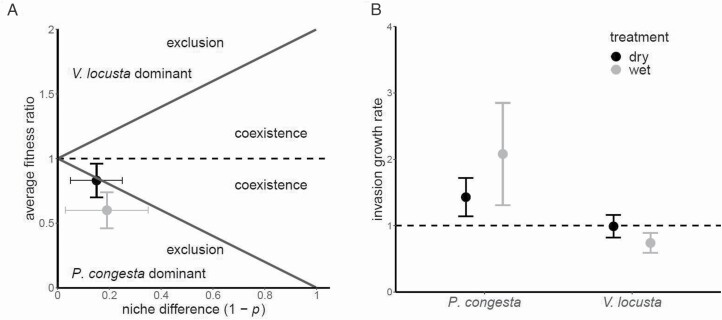
Coexistence predictions for *P. congesta* and *V. locusta* from a controlled experiment with dry and wet soil moisture conditions. (A) Coexistence is possible when niche overlap (ρ) is low, i.e. when ρ approaches 0 (or when niche difference (1 − ρ) approaches 1), and the fitness ratio (κ) is relatively even between species, i.e. when κ approaches 1, specifically when ρ < κ < 1/ρ. Here, fitness ratios >1 represent a *V. locusta* competitive advantage, while fitness ratios <1 represent a *P. congesta* competitive advantage. Error bars indicate standard errors. (B) Coexistence outcome can also be described using the low-density population growth rates of competing species. Species are expected to coexist when both species can invade and increase in abundance in a patch dominated by the opposing species, i.e. when both species display low-density invasion growth rates >1 (dashed line). Error bars indicate standard errors. **See****Equations 2****–****8**, **Supporting Information-Supplemental Methods** for details on parameter estimation.

The relative values of niche overlap (ρ) and the fitness difference ratio (κ _*j*_/κ _*i*_) predict that these species should not coexist, and that *P. congesta* will exclude *V. locusta* in both wet and dry conditions over the long run (Fig. 3). ρ was relatively large in both treatments, that is, close to 1 (Table 1); the niche difference (1 − ρ) was close to 0 (Fig. 3A). κ _*j*_/κ _*i*_ is a ratio of the product of the demographic potential and competitive response of each species. The demographic ratio changed significantly between treatments, favouring *V. locusta* in the dry treatment but *P. congesta* in the wet treatment (Table 1). The competitive response ratio changed significantly between treatments, from favouring *P. congesta* in the wet treatment to even more heavily favouring *P. congesta* in the dry treatment (Table 1). The opposing directions of the changes in demographic potential and competitive response resulted in a static average fitness ratio in favour of *P. congesta* in both the dry and wet treatments (Table 1; Fig. 3A). Average fitness differences that favour *P. congesta* over *V. locusta* combined with low niche difference project that *P. congesta* should competitively exclude *V. locusta* over the long run in both sets of environmental conditions, although the dry treatment estimates are within a margin of error of coexistence.

Coexistence can also be predicted if both species demonstrate the ability to invade and increase in abundance when rare, that is when the low-density invasion population growth is >1. With invasion growth rates above 1 in both wet and dry conditions, *P. congesta* could invade patches of equilibrium density *V. locusta* in both treatments (Fig. 3B; low-density invasion growth rates for *P. congesta* in wet treatment: 2.08 (SE = 0.77) and dry: 1.43 (SE = 0.29)). *Valerianella locusta* has the potential to invade in dry conditions (invasion growth rate above 1), but not in wet conditions (Fig. 3B; low-density invasion growth rate in wet: 0.74 (SE = 0.15) and in dry: 0.99 (SE = 0.17) in the dry treatment).

### Field density-dependence and environmental conditions

**Figure 4.**
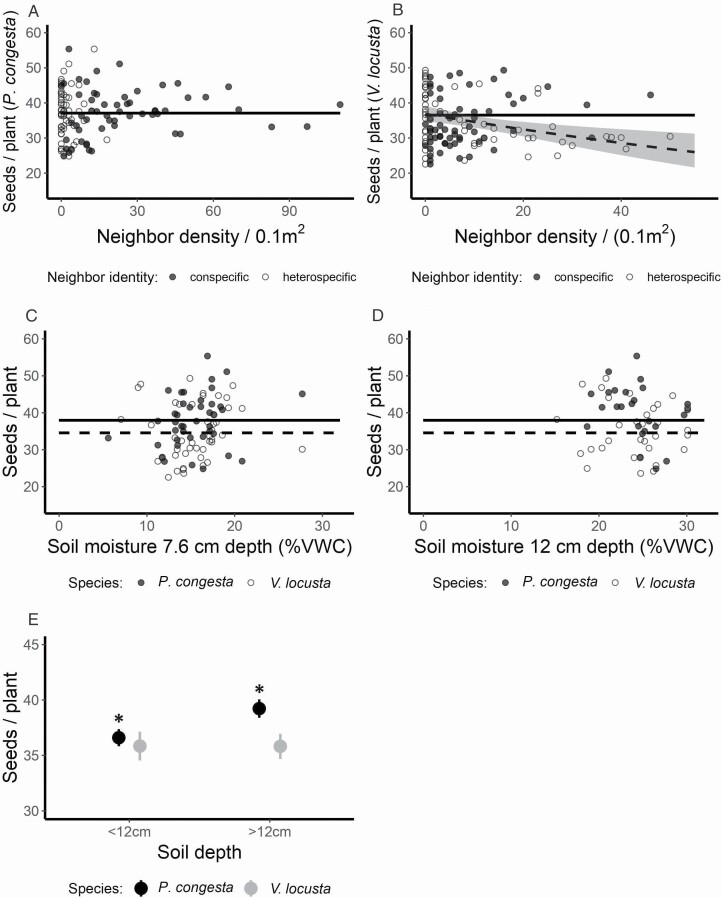
Relationships between predicted seed production and neighbour density and variation in environmental composition in natural plant communities. Effect of intraspecific (filled circles, solid trend lines) and interspecific (open circles, dashed trend lines) competition on predicted fecundity of (A) *P. congesta* and (B) *V. locusta* with 95 % CI for significant trend. Relationship between predicted seed production and (C) soil moisture at 7.6 cm depth, and (D) soil moisture at 12 cm depth for *P. congesta* (filled circles, solid trend lines) and *V. locusta* (open circles, dashed trend lines). (E) Mean and SE for *P. congesta* (black) and *V. locusta* (grey) growing in soils shallower or deeper than 12 cm.

In field conditions, we found that *P. congesta* fecundity did not decline with increasing intraspecific or interspecific densities (Fig. 4A), while *V. locusta* fecundity declined with increasing interspecific densities ($\chi_{1}^{2}$ = 5.78, *P* < 0.02) but not with increasing intraspecific densities (Fig. 4B).

Soil moisture had no effect on predicted seed production at either a depth of 7.6 cm (Fig. 4C) or 12 cm (Fig. 4D). These species exhibited unique responses to soil depth (depth > 12 cm versus < 12 cm): seed production increased with deeper soils in *P. congesta* ($\chi_{1}^{2}$ = 6.44, *P* = 0.01), but did not change in *V. locusta* ($\chi_{1}^{2}$ = 1.07, *P* = 0.30) (Fig. 4E). Soil moisture varied within and between plots, and across transects, with more than half of the variation observed at the smallest spatial scale, within 1-m^2^ plots **[see****Supporting Information—Fig. S2****]**. We did not find any significant interactions between any combination of environmental condition and neighbour density on fecundity.

## Discussion

To understand how invasion success and impact of an introduced plant species is mediated by a closely related native plant species, we used a coexistence framework to make long-term predictions about competitive interaction outcome. We further examined whether the niche and fitness differences that underlie long-term coexistence in direct-interaction scales were environmentally dependent, potentially enabling community-scale coexistence despite local exclusion under some circumstances. We found that even though the underlying components of niche and fitness differences showed dependence on environmental variation, the ultimate outcome was that native *P. congesta* is expected to competitively exclude introduced *V. locusta* across environmental conditions. Here we discuss factors contributing to our results, potential limitations and implications for invasion impact.

### Competitive dominance of native *P. congesta* over introduced *V. locusta*

Our experimental results predict that *P. congesta* will tend to exclude *V. locusta* due to a slight fitness inequality combined with modest niche difference (Fig. 3). In field conditions, we observed that *P. congesta* does not reduce its own fecundity with increasing density, but does have a negative density-dependent effect on the fecundity of *V. locusta* (Fig. 4A and B). Since field measurements of competitive response were taken across the range of environmental variation that these species experience, these field results expand on our experiment by suggesting that *P. congesta* is competitively dominant across most field conditions. The weaker relative response of *P. congesta* to neighbour density in both our experimental treatments and across the field plots can contribute to a pattern of dominant occurrence and abundance relative to *V. locusta* (Hart *et al.* 2018). We infer that the competitive dominance of native *P. congesta* over introduced *V. locusta* at the scale of direct interactions contributes to the inverse pattern of abundance that motivated us to explore the competitive interactions between these species (Fig. 1).

Low-density invasion growth rates are an alternative way to consider these data, and suggest that *P. congesta* can increase in population size when moving into patches of *V. locusta* or after experiencing a reduction in abundance. Positive low-density population growth rates of *V. locusta* are especially unlikely when soil moisture is higher (Fig. 2B). It has long been understood that the availability of water or other limiting resources interacts with competition in general (e.g. Reader and Best 1989; Going *et al.* 2009). However, the mutual invasibility criterion that we used allows us to make the distinction that although water availability interacts uniquely with intraspecific and interspecific competition in this species pair, this interaction does not change long-term population-level predictions for these species.

Studies of coexistence that manipulate water availability in similar annual plant communities in Australian and Mediterranean grassland communities show inconsistency in environmental dependency, whether the experiments were in pots (Germain *et al.* 2016; Staples *et al.* 2016) or the field (Matias *et al.* 2018; Wainwright *et al.* 2019). While the overall niche and fitness differences remained relatively the same in both ours and one other controlled pot experiment (Staples *et al.* 2016), the others found that niche and fitness differences increased, decreased or remained the same with no clear pattern across species pairs and watering treatments (Germain *et al.* 2016; Wainwright *et al.* 2019), or that niche and fitness differences both decreased in response to decreased water availability (Matias *et al.* 2018). Alongside our study, these results highlight that water availability—due to either natural within- and between-community variation, extreme environmental events or long-term climate change—does not predictably influence long-term coexistence. In the above studies, as well as in our own, water availability is considered an important limiting resource across the plant community; however, we do not know whether this resource was truly limiting for either or both species. If a resource is not strongly limiting, then availability of that resource should have negligible impact on niche and fitness differences. Therefore, the inconsistency of coexistence outcomes in response to water availability across studies may result from inconsistency in the degree to which water is a limiting resource.

We did not find any interaction between soil moisture and competition in the field, supporting our experimental findings that coexistence is not strongly dependent on soil moisture. However, our field data indicate that *P. congesta* fecundity increases in deeper soils (Fig. 3E). Soil depth may be a better measure of soil moisture in the field versus our single point samples of %VWC because deeper soils exhibit higher temporal stability of soil moisture (Penna *et al.* 2013), possibly better reflecting average soil moisture across the growing season. If deeper soils indicate higher soil moisture, then the increased fecundity of *P. congesta* on deep soils mirrors the increased competitive advantage of *P. congesta* in our experimental wet treatment. Deeper soils may also reflect higher availability of nutrient resources and contribute to plant community structure in this savanna system (MacDougall and Turkington 2005).

Contrary to expectations that introduced species that establish and spread should limit native species, our results project that a common introduced species will not significantly impact the abundance of a closely related and functionally similar native species. Instead, our results suggest that native *P. congesta* limits the ability of introduced *V. locusta* to increase in abundance in neighbourhood patches where these species co-occur. Because the invasion of *V. locusta* has little impact on a species with close phylogenetic relationship and overlap in functional traits and life history—a species pair where we would expect the highest degree of niche overlap (HilleRisLambers *et al.* 2012)—we conclude that invasion of introduced *V. locusta* has low impact on these plant communities as a whole. This result of a native species limiting an invader because of high niche overlap and a fitness inequality provides a mechanistic example of a process that contributes to the well-accepted phenomenon that diversity of native residents reduces success of non-native invasions (Levine *et al.* 2004; Maron and Marler 2007). Our results align with the result across many studies that species interactions typically do not completely repel invasion but still may constrain the abundance of invasive species once they have established (Levine *et al.* 2004).

Investigating other elements of demographic performance that we did not measure might be important to gain a more complete understanding of how patterns in these species’ abundances arise. In many systems, populations are more limited by space for seeds to germinate rather than by the production of seeds (Grubb 1977). Germination rates in the field may limit the ability of *P. congesta* to spread locally to exclude *V. locusta*, especially given the abundance of large perennial non-native grasses and other introduced species that cover the background matrix and likely limit space for germination (MacDougall and Turkington 2006). Large non-native perennial grasses, which are known to limit the abundance and reproduction of many species in this system and which become most dominant in patches with deep soil and high water availability (MacDougall and Turkington 2005), may also influence the competitive outcome and invasion dynamic of our focal species. Our field data were collected largely from patches that were not heavily dominated by non-native perennial grasses. Although it is likely that these grasses would affect *P. congesta* and *V. locusta* through impacts on the demography of each species and not by changing the intrinsic interaction between them (Godoy *et al.* 2017), we expect that these large non-native perennial grasses outcompete and exclude both of these small annual species in patches with the deepest soils and highest water availability.

### Coexistence at the community scale despite competitive exclusion at the direct-interaction scale

Our finding that this species pair co-occurs at the community scale (Fig. 1) despite the failure to meet direct-interaction coexistence conditions (Fig. 2), aligns with other recent studies of coexistence in annual plant communities. Only a minority of co-occurring species are predicted to coexist in similar grassland systems in California (Kraft *et al.* 2015), Spain (Matias *et al.* 2018) and Australia (Staples *et al.* 2016; Wainwright *et al.* 2019). Together, these results highlight that coexistence mechanisms that operate beyond the scale of individual interactions and rely on spatial or temporal heterogeneity—such as variation in resources and soil at the site scale (e.g. Eckhart *et al.* 2017) or temporal storage effects (Angert *et al.* 2009)—make a large contribution to the co-occurrence pattern at the community scale. We hypothesize that spatial mechanisms may promote coexistence of these species, explaining the continued presence of introduced *V. locusta* despite its competitive inferiority. We found that the gap between fitness and niche differences decreased in the dry treatment in our experiment (Fig. 3) and that *P. congesta* seed production decreases in shallower soils (Fig. 4E). If these trends were to continue, coexistence via a spatial storage mechanism might be enabled by allowing *V. locusta* to persist by maintaining positive low-density growth rates in the patches with driest or shallower soils within a community and then dispersing across the community from these patches. We expect this might be possible in natural populations because the experimental wet treatment was wetter than field surveys while the dry treatment was near the average soil moisture conditions observed **[see****Supporting Information—Table S1****and****Fig. S2****]**. Alternatively, other resources that are more limiting in this system, such as nitrogen (MacDougall and Turkington 2005), may enable a spatial storage effect if they force a stronger environmental dependency.

The coexistence of these species at our field sites might also be promoted through spatially dependent growth-density covariance, i.e. low spatial autocorrelation in the environment that increases the likelihood for species coexistence in a given area because it enables species to aggregate in their own favourable conditions, especially when dispersal distances are less than the spatial grain size of environmental heterogeneity (Hart *et al.* 2017). We found that the largest amount of variation in water availability arises from within the smallest spatial grain size that we considered, within 1-m^2^ plots **[see****Supporting Information—Fig. S2****]**, meaning that there is low spatial autocorrelation. Quantifying dispersal distances rates would be necessary to determine whether these species are able to segregate into their own favourable habitat patches (Hart *et al.* 2017). Data on the dispersal capability of *P. congesta* suggest that most seeds fall within 10 cm of the parent plant (Trowbridge 2015). If these short dispersal distances generally do not exceed the grain size of environmental heterogeneity, then growth-density covariance would act as another spatially dependent mechanism to enable coexistence at the scale of an entire community, despite an inability to coexist in certain patches within the plant community.

## Conclusions

Previous use of the coexistence framework to describe invasion mechanisms suggests that low-impact invasions should arise from high niche differences that couple with little or no invader fitness advantages (MacDougall *et al.* 2009; Lai *et al.* 2015). Low-impact invasion in our system arises through an alternative scenario, where an introduced species with high niche overlap is only maintained through coexistence mechanisms that operate beyond the direct-interaction scale. Low-impact invaders that possess a high niche difference would result in a low impact across the community. In contrast, low-impact invasions that emerge through spatial or temporal mechanisms as is inferred in our system, have the potential to result in introduced species that exhibit competitive exclusion and local extinction of natives in certain places or at certain times if environmental dependency shifts coexistence. Given these different repercussions it is important to differentiate between these separate mechanisms of low-impact invasion. In our system this may be especially important if fitness differences extend towards favouring the introduced *V. locusta* in even drier conditions than we tested because future climate projections anticipate warmer and drier summers (Bachelet *et al.* 2011).

By providing a quantitative link between a short-term empirical study and population-level predictions, our results provide mechanistic underpinning for the general finding that native species diversity reduces establishment and spread of introduced invaders; presence of species in the native community with high niche overlap may constrain an introduced species at the scale of direct interactions. Taken alongside other studies that recognize that few co-occurring species pairs are suspected to coexist according to quantified niche and fitness differences, this study emphasizes that coexistence mechanisms that operate beyond the scale of direct interaction are necessary to maintain much of the species diversity that is observed across community scales.

## Supporting Information

The following additional information is available in the online version of this article—

**Supplemental Methods**

**Table S1.** Soil moisture conditions for competition experiment.

**Table S2.** Model selection for seed production in *P. congesta* and *V. locusta*.

**Figure S1.** Experimental design.

**Figure S2.** Proportion of variance in soil moisture at field sites.

plaa045_suppl_Supplementary_Material

## Data Availability

All data and corresponding program code is permanently archived with Knowledge Network for Biocomplexity (KNB, https://knb.ecoinformatics.org/view/urn:uuid:b70775a8-ae68-43bc-83f2-ec49c37bb7be).
