# Supplementary material for: A native annual forb locally excludes a closely related introduced species that co-occurs in oak-savanna habitat remnants
Source: AoB Plants. 2020 Aug 25;12(5):plaa045. doi: 10.1093/aobpla/plaa045 (PMC7532728; doi:10.1093/aobpla/plaa045)

Supporting Information, Methods

Growing Condition Details

For both the germination rates and the density gradient, we planted seeds in Sunshine Mix #1 (Terralink) soil medium on March 10th - 13th, 2017. We used 7.6 litre planting pots with a diameter of 22.9 cm, depth of 22.9 cm and a 0.041 m^2 surface area. We obtained *P. congesta* seeds from Saanich Native Plants (Victoria, BC) and *V. locusta* seeds from Salt Spring Seeds (Salt Spring Island, BC). To ensure at least one individual of desired species identity at the center of the pot for data collection, we sowed three focal individual seeds around a marker at the center of the pot. When one of these seeds germinated, the remaining two seeds or germinants were immediately removed from the marker area to eliminate any interaction. The seeds of both species were sown directly on the surface of the soil, as recommended for *P. congesta* (Saanich Native Plants, personal communication; Young-Mathews 2012). Bird netting was placed over the pots to avoid seed predation.

Following the germination period, the pots were arranged on a 10 m x 8 m watering grid. Pots were placed in rows with 23 cm spacing and surrounded with a 15 cm mulch layer to moderate temperature and moisture fluctuations. Water inputs were supplied via Netafim pressure compensated spray stakes (Southern Drip Irrigation), with flow rates of 12.1 litres/hour (recommended by manufacturer for pots of 3.8 - 19 litre size) and a spray radius of ~25 cm. Pots were rotated randomly through the watering grid (within wet or dry treatment blocks) weekly to reduce any effects of small variation in spray stake water delivery. A temporary fence was constructed around the experiment area to deter herbivory from geese or other large animals.

All pots from both treatments were exposed to natural outdoor rainfall conditions from the start of the experiment until June 1, 2017, near when natural precipitation conditions shifted

from near daily and constant to infrequent and ephemeral. Precipitation patterns generally become more infrequent and variable later in the growing season in the region's Mediterranean dry summer climate (Lea 2006). At this date, plants were separated into wet and dry treatment groups. The semi-exposed aboveground pots dry more quickly than the soil, and thus artificial watering was necessary to maintain soil moisture conditions that would occur at ground level without any rainfall. Plants in the dry treatment received no water for 10 days, and then were maintained with 5 minutes of water every other day until the end of the experiment. Plants in the wet treatment received no water for 5 days, and then were maintained with 12 minutes of water every other day from until the end of the experiment. We added 0.25 liters of 100ppm, 20-8-20 fertilizer solution to all pots on June 5th, 2018, to replenish soil nutrients.

To test the differences in soil moisture between the two treatments, we measured the % VWC at 7.6 cm depth on an afternoon in early July on a day where the plants received water and again on the following day when the plants did not receive water. We compared the mean % VWC for the dry and wet treatment groups using a two-sample t-test and found that the mean % VWC was significantly different both on a watering day and on a no water-input day (Supplementary Table 2).

Analysis of Resource Competition Experiment

To determine whether *P. congesta* and *V. locusta* can coexist in direct competition, we used data on vital rates in the assembled wet and dry treatment communities to parameterize population models. These models estimate both population growth rates in the absence of competition (λ), and the influence of neighbor density and identity on population growth rates ($\alpha_{\text{conspecifc neighbors}}$ and $\alpha_{\text{heterospecifc neighbors}}$). This approach allowed us to translate measurements of

individual fecundity from an annual growing season into predictions for the competing populations over the long term (Hart et al. 2018).

The population dynamics of an annual plant species with no seed bank can be described as (Chesson 1994):

Equation 1: $\frac{N_{i,t+1}}{N_{i,t}} = g_i F_i$.

Here, $N_{i,t}$ represents the number of ungerminated seeds for species *i* at time *t*. The per capita growth rate for the population $((N_{i,t+1})/(N_{i,t}))$ is expressed as a function of F_i, fecundity or the viable seeds produced per germinated individual, weighted by the average germination of the species, g_i (Chesson 2000, Godoy and Levine 2014). To determine how species affect the performance of one another, the per-germinant fecundity, F_i, can be expanded into a function that considers the effect of competing individuals in the system (Chesson 2000, Godoy and Levine 2014):

Equation 2: $F_i = \frac{\lambda_i}{1+ \alpha_{ii} g_i N_{i,t} + \alpha_{ij} g_j N_{j,t}}$.

Here, λ_i represents the per-germinant fecundity in the absence of competition. F_i decreases when neighboring individuals of either the same species or another species negatively impact the performance of plants. These interaction coefficients are represented by α_{ii} (intraspecific interaction coefficient for species *i*), and α_{ij} (interspecific interaction coefficient for species *i*). Larger values of α represent higher impacts of each neighbor on per capita seed production. We used fecundity data from each watering treatment in the experiment to fit Equation 2 and then estimated values of λ and α for both *P. congesta* and *V. locusta* using generalized nonlinear regression with a negative binomial distribution with gnlm (Swihart and Lindsey 2019). We fixed a common value of λ regardless of whether the competitors were intra-

or interspecific. We used boot (Canty and Ripley 2019) to determine mean estimates and standard deviations of λ and α values by bootstrap resampling the density-dependent seed production data (parameter R = 50).

These demographic measurements were then synthesized into estimates of the stabilizing niche differences and fitness differences that determine the outcome of competition (Chesson 2000, Godoy and Levine 2014). Niche overlap (ρ) is defined as:

Equation 3: $$\rho = \sqrt{\frac{\alpha_{ij}}{\alpha_{jj}} * \frac{\alpha_{ji}}{\alpha_{ii}}}\,.$$

The demographic ratio, which describes the degree to which one species produces more seeds per seed loss due to death or germination, is defined as:

Equation 4: $$demographic\ ratio = \frac{\lambda_j * g_j}{\lambda_i * g_i}\,.$$

The competitive response ratio, which describes the relative degree to which the two species retain their potential fecundity despite increased crowding from intra- and interspecific neighbors, is defined as:

Equation 5: $$competitive\ response\ ratio = \sqrt{\frac{\alpha_{ij}}{\alpha_{jj}} * \frac{\alpha_{ii}}{\alpha_{ji}}}\,.$$

Fitness differences (κ_i/κ_j) are defined as the product of the demographic ratio between species and the competitive response ratio:

Equation 6: $$\kappa_i/\kappa_j = demographic\ ratio * competitive\ response\ ratio\,.$$

Niche and fitness differences are functions based on combinations of parameter estimates with associated uncertainties. We used the propagate function with propagate (Spiess 2018) to carry errors in the parameter estimates of λ and α through composite variables to obtain confidence intervals for our niche and fitness difference estimates. Propagation confidence

intervals are calculated from the expanded uncertainties by means of the effective degrees of freedom. Finally, we considered coexistence conditions satisfied when:

Equation 7: $\rho < \kappa < \frac{1}{\rho}$

Given Equation 7, coexistence is most likely when niche overlap (ρ) is low, that is, when ρ approaches 0, and the fitness ratio (κ) is relatively even between species, that is, when κ approaches 1.

Coexistence can also be described using the low-density population growth rates of competing species; species are expected to coexist when both species can invade and increase in abundance in a patch dominated by the opposing species (Chesson 2000). We quantified the invasion growth rate of species *j* (occurring at a diminishingly low density) invading species *i* (occurring at its equilibrium population density) using the following equation (Hart et al. 2019):

Equation 8: $low\ density\ invasion\ growth\ rate\ (j) = \frac{\lambda_j}{1 + \alpha_{ji} * \frac{(\lambda_i - 1)}{\alpha_{ii}}}$.

Supplement References

Canty, A. and Ripley, B. 2019. boot: Bootstrap R (S-Plus) Functions. R package version 1.3-24.

Chesson, P. 1994. Multispecies competition in variable environments. *Theoretical Population Biology*, *45*: 227-276.

Chesson, P. 2000. Mechanisms of maintenance of species diversity. *Annual Review of Ecology and Systematics*, *31*: 343-366.

Hart, S.P. et al. 2018. How to quantify competitive ability. *Journal of Ecology*, *106*: 1902-1909.

Hart, S.P. et al. 2019. Effects of rapid evolution on species coexistence. *Proceedings of the National Academy of Sciences*, *116*: 2112-2117.

Godoy, O. and Levine, J.M. 2014. Phenology effects on invasion success: insights from coupling field experiments to coexistence theory. *Ecology*, *95*: 726-736.

Lea, T. 2006. Historical Garry oak ecosystems of Vancouver Island, British Columbia, pre-European contact to the present. *Davidsonia*, *17*: 34-50.

Spiess, A.N. 2018. propagate: Propagation of Uncertainty. R package version 1.0-6. https://CRAN.R-project.org/package=propagate.

Swihart, B. and Lindsey, J. 2019. gnlm: Generalized Nonlinear Regression Models. R package version 1.1.1. https://CRAN.R-project.org/package=gnlm.

Young-Mathews, A. 2012. Plant fact sheet for shortspur seablush (*P. congesta congesta*). USDA-Natural Resources Conservation Service, Plant Materials Center, Corvallis, OR

Supporting Information, Tables and Figures

Table S1: Soil moisture conditions for competition experiment.

	Day of watering	Day after watering
Mean % VWC (dry treatment)	20.3	18.3
Mean % VWC (wet treatment)	34.8	33.9
Degrees of freedom	30	30
t-statistic	5.09	6.01
P-value	< 0.01	< 0.01

Table S2: Model selection for seed production in *P. congesta* and *V. locusta*. Models are ranked by AIC, and best model chosen by lowest AIC value (bolded and starred).

Species	Fitness Proxies	AIC
P. congesta	plant height	**351***
	number of inflorescences + plant height	351.38
	plant height + main inflorescence size	352.98
	number of inflorescences + plant height + main inflorescence size	353.35
	number of inflorescences	357.99
	number of inflorescences + main inflorescence size	359.98
	main inflorescence size	360.06
V. locusta	number of inflorescences + plant height + main inflorescence size	**1892***
	number of inflorescences + plant height	1894.5
	plant height + main inflorescence size	1908.5
	plant height	1911.2
	number of inflorescences + main inflorescence size	1915.4
	number of inflorescences	1927.3
	main inflorescence size	2216.1

Table S3: Average monthly precipitation during the growing season (March through May) at Victoria International Airport, Victoria, BC. Field observations and controlled experiments were conducted in spring and summer of 2017.

**Year**	**Average Monthly Precipitation (mm)**
2000	43.3
2001	45.9
2002	60.0
2003	66.8
2004	30.2
2005	61.0
2006	41.9
2007	61.1
2008	38.2
2009	50.1
2010	60.6
2011	81.8
2012	53.6
2013	65.9
2014	67.6
2015	41.7
2016	47.3
**2017**	**85.1**
2018	40.3

Figure S1: Experiment Design. (A) *P. congesta* grown against a density-gradient background of conspecific neighbors or *V. locusta*. (B) *V. locusta* grown against a density-gradient background of conspecific neighbors or with *P. congesta*. These setups were conducted under either wet or dry conditions.

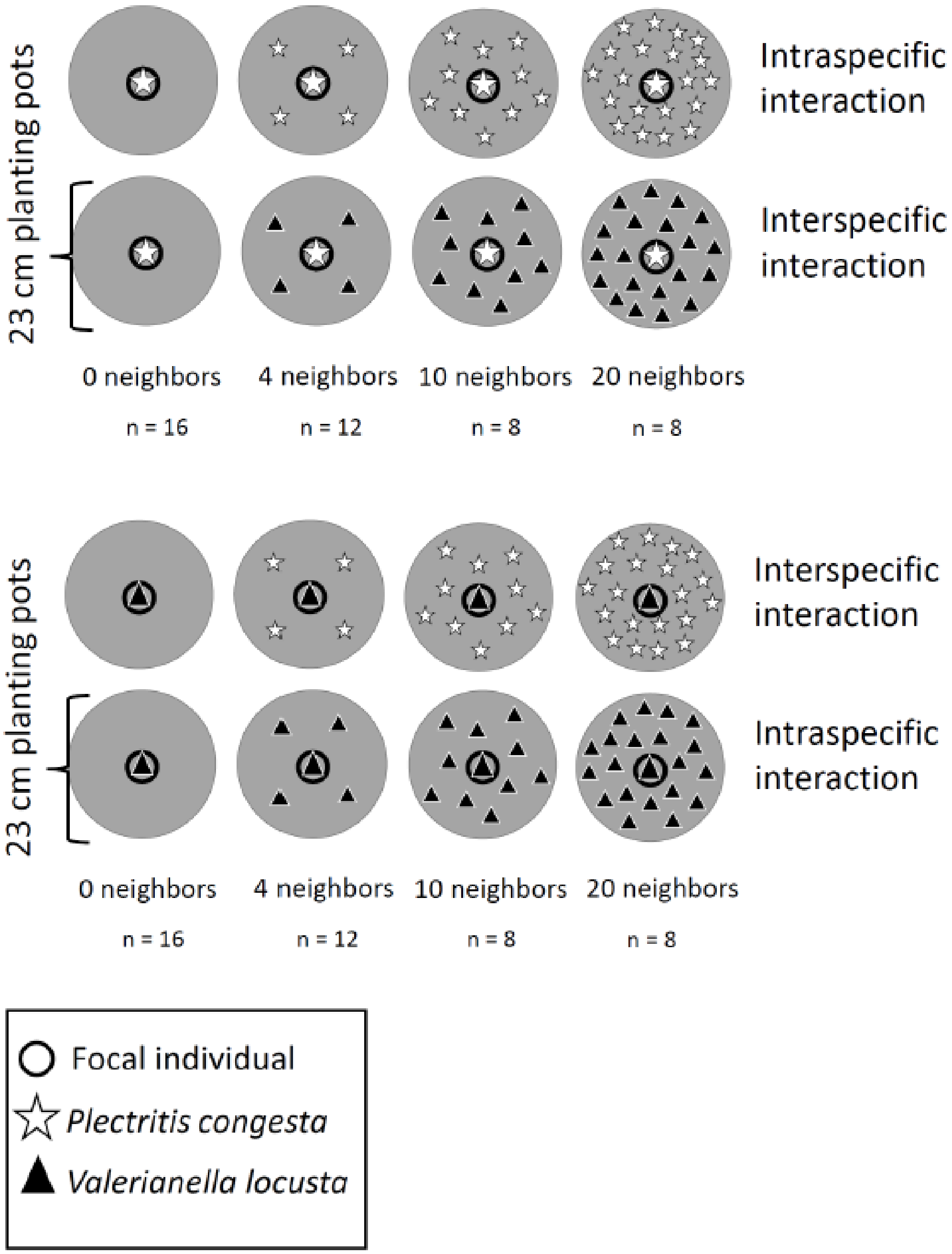

Figure S2: Proportion of variance in soil moisture at field sites arising from differences between transects, between plots, or within 1 m^2 plots. Black points indicate measurements taken at 7 cm depth and gray points indicate measurements taken at 12 cm depth. Error bars represent standard deviations.

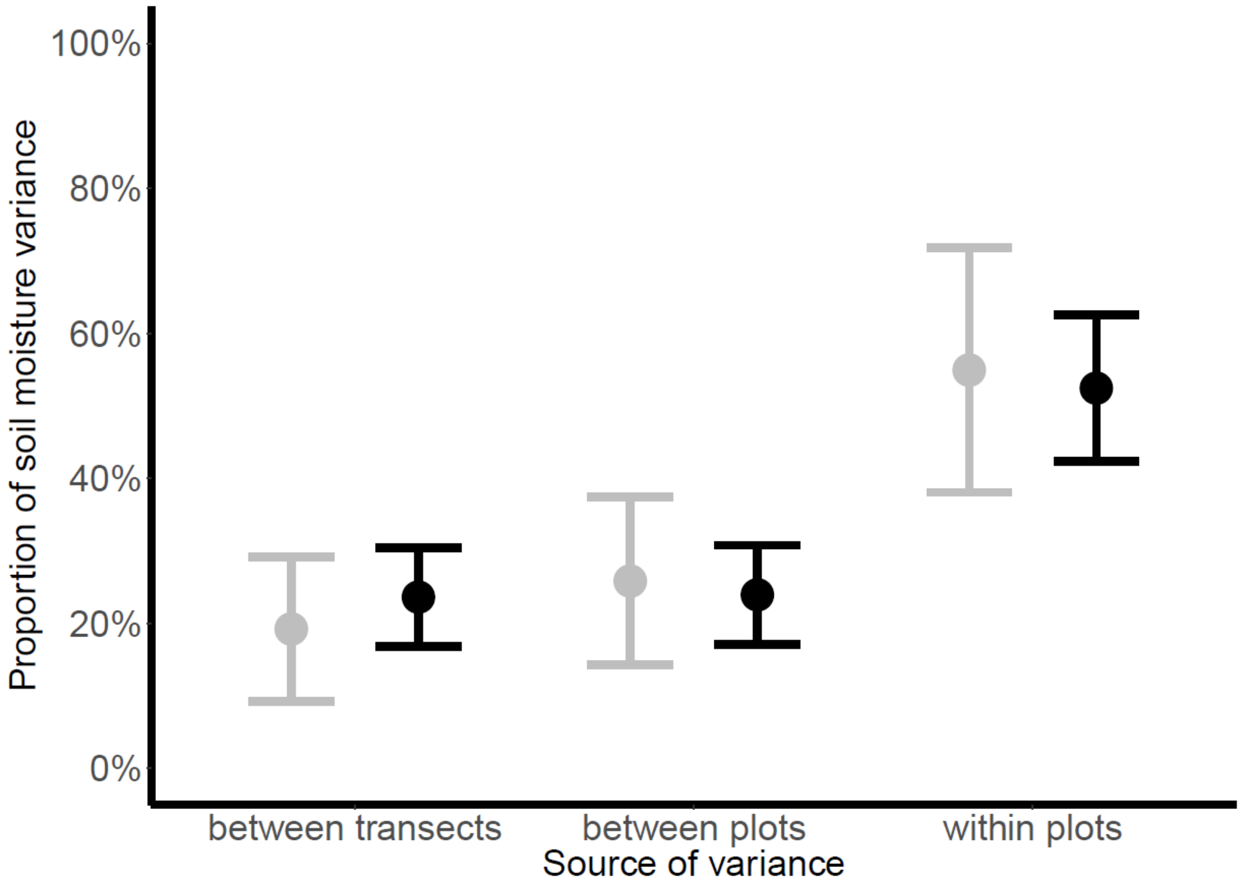

Supplement: plaa045_suppl_Supplementary_Material [file plaa045_suppl_supplementary_material.pdf]
